# Geniposide and Chlorogenic Acid Combination Improves Non-Alcoholic Fatty Liver Disease Involving the Potent Suppression of Elevated Hepatic SCD-1

**DOI:** 10.3389/fphar.2021.653641

**Published:** 2021-05-04

**Authors:** Cheng Chen, Xin Xin, Qian Liu, Hua-Jie Tian, Jing-Hua Peng, Yu Zhao, Yi-Yang Hu, Qin Feng

**Affiliations:** ^1^ Institute of Liver Diseases, Shuguang Hospital Affiliated to Shanghai University of Traditional Chinese Medicine, Shanghai, China; ^2^ Shanghai Key Laboratory of Traditional Chinese Clinical Medicine, Shanghai, China; ^3^ Key Laboratory of Liver and Kidney Diseases, Shanghai University of Traditional Chinese Medicine, Ministry of Education, Shanghai, China

**Keywords:** non alcoholic fatty liver disease, metabolic associated fatty liver disease 1, stearoyl-CoA desaturase-1, *de novo* lipogenesis, geniposide, chlorogenic acid

## Abstract

**Background: **Non-alcoholic fatty liver disease (NAFLD), characterized by the excessive accumulation of hepatic triglycerides (TGs), has become a worldwide chronic liver disease. But efficient therapy keeps unsettled. Our previous works show that geniposide and chlorogenic acid combination (namely the GC combination), two active chemical components combined with a unique ratio (67.16:1), presents beneficial effects on high-fat diet-induced NAFLD rodent models. Notably, microarray highlighted the more than 5-fold down-regulated SCD-1 gene in the GC combination group. SCD-1 is an essential lipogenic protein for monounsaturated fatty acids’ biosynthesis and serves as a key regulatory enzyme in the last stage of hepatic *de novo* lipogenesis (DNL).

**Methods: **NAFLD mice model was fed with 16 weeks high-fat diet (HFD). The pharmacological effects, primarily on hepatic TG, TC, FFA, and liver enzymes, et al. of the GC combination and two individual components were evaluated. Furthermore, hepatic SCD-1 expression was quantified with qRT-PCR, immunoblotting, and immunohistochemistry. Finally, the lentivirus-mediated over-expressed cell was carried out to confirm the GC combination’s influence on SCD-1.

**Results: **The GC combination could significantly reduce hepatic TG, TC, and FFA in NAFLD rodents. Notably, the GC combination presented synergetic therapeutic effects, compared with two components, on normalizing murine hepatic lipid deposition and disordered liver enzymes (ALT and AST). Meanwhile, the robust SCD-1 induction induced by HFD and FFA in rodents and ALM-12 cells was profoundly blunted, and this potent suppression was recapitulated in lentivirus-mediated SCD-1 over-expressed cells.

**Conclusion: **Taken together, our data prove that the GC combination shows a substantial and synergetic anti-lipogenesis effect in treating NAFLD, and these amelioration effects are highly associated with the potent suppressed hepatic SCD-1 and a blunted DNL process.

## Introduction

Non-alcoholic liver disease (NAFLD), newly named metabolic dysfunction-associated fatty liver disease (MAFLD), is the leading chronic liver disease, affecting approximately 1.7 billion individuals worldwide (Eslam et al., 2020; ). Nowadays, NAFLD and its complications pose a tremendous health burden not only in western countries but also in developing countries, for instance, in China (Zhou et al., 2020). NAFLD is characterized by the excessive accumulation of triglycerides (TGs) in hepatocytes without alcoholic abuse (Rinella, 2015). The mechanism of NAFLD is still not fully understood. The “multiple hits” is considered an influential contributor to severe NAFLD and NASH development. Notably, hepatic *de novo* lipogenesis (DNL) is a fundamental biosynthetic pathway within liver, leading to the lipids secreted and stored by hepatocytes, which involves a sequence of fatty acid synthetase. Among these fatty acid synthetases, SCD-1 is a terminal enzyme controlling the DNL, and the induction of SCD-1 is highlighted to be a key player in the pathogenesis and progression of NAFLD (Jensen-Urstad and Semenkovich, 2012; Sanders and Griffin, 2016).

Our previous research has identified that the recipe composed of atractylodes macrocephala polysaccharide, chlorogenic acid, and geniposide (namely the ACG recipe), derived from traditional Chinese medicine Qushi Huayu Decoction (QHD) (Tang et al., 2013; Feng et al., 2013; Peng et al., 2018; Leng et al., 2020; Feng et al., 2017), presents the therapeutic effects on NAFL rodent by improving hepatic lipid deposition and inflammation (Meng et al., 2016). Atractylodes macrocephala polysaccharide (A), chlorogenic acid (C), and geniposide (G), these three components were identified as the primary active ingredients in QHD (Meng et al., 2016), and they were randomly but in a formulaic way selected and combined by uniform design. For further simplifying these three natural products compounded ACG recipe. These three components were also screened by the consistent design. Finally, the regression equation Y = 71.966−19.798 × X4 + 18.687 × X1 × X4 isolated from *artemisia capillaries Thunb*, might exhibit the most potent protective effects against hepatic lipid deposition. This unique combination of geniposide and chlorogenic acid was named as the GC combination.

Notably, the GC combination presents multi-therapeutic effects on improving NAFLD by reducing gut inflammation, evaluating gut barrier function, and anti-oxidative stress in NAFL rodent (Peng et al., 2018; Feng et al., 2017). However, whether the GC combination is directly involved in the hepatic lipid synthesis process and its specific molecular mechanism still keeps unclear. Preliminarily, microarray assay reported that stearoyl-CoA desaturase-1 (SCD-1) hits the No. 1. among the biomarkers influenced by the GC combination, and it was surprisingly five times down-regulated. SCD-1 is a crucial enzyme for monounsaturated fatty acids’ biosynthesis and serves as a key regulatory enzyme in the last stage of hepatic DNL (Liu et al., 2017a; Kumar et al., 2020). Its primary substrates C16:0 and C18:0, and products palmitoleic acid (C16:1) and oleic acid (C18:1) are the most abundant fatty acids in TGs, cholesterol esters, and membrane phospholipids (Poloni et al., 2015). In humans and rodents, SCD-1 is mainly expressed in liver and adipose tissue. SCD-1 deficiency protects against high-fat-, high-carbohydrate-, and leptin deficiency-induced obesity and hepatic steatosis (Miyazaki et al., 2009). On the contrary, SCD-1 level was aggravated 10- to 15-fold in animal models after fed with a high-glucose and high-fat diet (Basciano et al., 2009). Simultaneously, SCD-1 inhibitors represent a potential treatment for NAFLD (Rotman and Sanyal, 2017).

Here, we evaluate the pharmacological effects of the GC combination in the NAFLD model. We primarily focus on the hepatic index correlated with lipid and glucose metabolism. Meanwhile, based on the microarray assay results, SCD-1 is expected as a potential therapeutic target of the GC combination for ameliorating hepatic lipid accumulation in NAFLD. We further explore the effect of the GC combination on SCD-1 in free fatty acids induced and lentivirus transfected SCD-1 over-expressed cells. Our data show the apparent amelioration effects of GC combination by removing hepatic lipid accumulation on HFD induced fatty liver. Moreover, the GC combination presents a robust suppression effect on hepatic SCD-1 *in vivo* and *in vitro*, which highly blunts the DNL process. Since SCD-1 and DNL play an essential role in lipid metabolism in NAFLD, suppressed SCD-1 expression could be a reason to clarify the therapeutic effects of the GC combination.

## Materials and Methods

### Drug Preparation

Geniposide (drug purity was 98%, lot number CY101018) derived from *Gardenia jasminoides Ellis*, Chlorogenic acid (drug purity was 98%, lot number GY0900705) derived from *artemisia capillaries Thunb*, all drugs were purchased from Shanghai Winherd medical technology Co., Ltd., Shanghai, China. The high-performance liquid chromatography (HPLC) analysis of two drugs is presented in Supplementary Figure S1, and the method was described previously (Feng et al., 2013). The ratio of geniposide (G) and chlorogenic acid (C) in the GC combination is 67.16:1.

### Animal Experimental Design

The protocols for animal studies were reviewed and approved by the Animal Studies Ethics Committee of Shanghai University of Traditional Chinese Medicine (NO. PZSHUTCM190621014). The rat experiment was identified before (Meng et al., 2016). Here, Fifty 6 week-old male C57BL/6J mice were purchased from the Shanghai Laboratory Animal Center (Shanghai, China). Animals were maintained at room temperature on a 12h:12h light-dark cycle in the animal center of the Shanghai University of Traditional Chinese Medicine.

After acclimation for 1 week, Fifty C57BL/6J mice were randomly divided into normal diet group (ND, *n* = 10) and high-fat diet group (*n* = 40). The mice in the high-fat diet group were fed a 60.0% kcal fat diet (D12492i, Research Diets, New Brunswick), and the mice in the normal diet group were fed a 10.0% kcal control diet (D12450B, Research Diets, New Brunswick). After 12 weeks on the respective diets, mice in the HFD group were further randomized into four groups: HFD group (HFD), HFD plus the GC group (HFD + GC), HFD plus the G (HFD + G), and the C group (HFD + C), 10 mice in each group. In the following 4 weeks, the mice were administrated with different drugs by gavage once per day. The G (90 mg/kg per day), the C (1.34 mg/kg per day) and, the GC combination [(90 mg G + 1.34 mg C)/kg per day] were dissolved in drinking water, and HFD group mice received an equal volume of drinking water. All the animals were sacrificed for tissue collection at the end of the 16th week.

### Microarray Data Analysis

RNA samples were subjected to transcriptome analysis using Affymetrix Rat Genome 230 2.0 Array. Microarray data is available at PubMed under accession number GSE87432. The MicroArray Suite 5 (MAS5) was used for data normalization in consideration that MAS5 makes use of the “Perfect Match – Mismatch” signals and that expression values determined from MAS5 are not on a logarithmic scale. Pathway analysis was performed with the Ingenuity Pathways Analysis (IPA; Ingenuity Systems, Inc., Redwood City, CA, www.ingenuity.com). Input lists included DEGs between experimental groups (False Discovery Rate < 0.05, *t*-test) (Feng et al., 2017).

### Measurement of Triglyceride, Total Cholesterol, and Free Fatty Acid Content in Liver Tissue

The wet liver (approx. 100 mg) was homogenized in 1.5 ml of ethanol-acetone (1:1) and kept at 4°C for 12 h. Afterward, the sample was centrifuged at 3,000 rpm for 15 min, and the suspension was collected for the determination of triglyceride content and total cholesterol by using biochemistry assay kits (Dongou Biology Technique Co. Ltd., Zhejiang, China). To measure the amount of hepatic FFA content, we use 100 mg wet liver tissue of each sample. The samples were homogenized in 0.9 ml saline and centrifuged at 3,500 rpm for 15 min. The suspension was collected for the determination of FFA content by using commercial kits (Nanjing Jiancheng Institute of Biotechnology, Nanjing, China) according to the manufacturer’s instructions.

### Serum Biochemical Parameter Analysis

The analysis of alanine aminotransferase (ALT) and aspartate aminotransferase (AST) was measured by commercial kits (Nanjing Jiancheng Institute of Biotechnology, Nanjing, China) according to the manufacturer’s instructions. Fasting blood glucose (FBG) was determined with a blood glucose meter (Roche diagnostic GmbH, Germany). Fasting insulin (FI) level was measured using the Mouse Insulin ELISA (ALPCO, America). The homeostasis model assessment of basal insulin resistance (HOMA-IR) was calculated using the formula FBG (mM) × FI (mM)/22.5.

### Histological Examination and Assessment

Sections of the liver samples (4 μm thick) or the frozen liver tissues (5 μm thick) were stained with hematoxylin-eosin (H and E) or oil red O. They were examined under the light microscope (Olympus Medical Systems Corp, Tokyo, Japan) (Tang et al., 2013). The cells were washed with PBS twice, fixed with 10% formalin at room temperature for 30 min, and stained with a freshly prepared working solution of Oil-Red O (Sigma, St. Louis, MO, United States) at room temperature for 30 min. The cells were observed in an Olympus microscope and documented. The concrete operating steps were followed by the previous studies (Feng et al., 2013).

### Cell Culture and Experimental Design *In Vitro*

AML-12 cells established from mouse hepatocytes (CD1 strain, line MT 42) were purchased from the Institutes of Biochemistry and Cell Biology, Shanghai Institutes for Biological Sciences, CAS, Shanghai, China. The cells were maintained in complete growth Dulbecco’s Modified Eagle’s Medium/Ham’s F-12 growth medium (DMEM/F-12 medium, GIBCO BRL) 1:1 with 10% fetal bovine serum (FBS; GIBCO-BRL, Grand Island, NY), ITS (1.0 mg/ml insulin, 0.55 mg/ml transferring, 5 ug/ml selenium, Sigma) and 40 ng/ml dexamethasone, at 37°C in a humidified incubator with 5% CO_2_.

The experiment started when the cells grew to 60–70% confluence. Cellular steatosis was induced by a mixture of 0.3 mM FFA (the ratio of oleate to palmitate is 2:1) in DMEM/F-12 containing 10% FBS for 24 h. Then the cells were divided into different groups as control group (CO), FFA group (FFA), FFA +200 μm GC group (FFA + GC) group. After the cells were incubated with respective drugs for another 24 h, the cells were stained with Oil-Red O and imaged. The cytotoxicity of the GC combination was measured by CCK-8 kit (Sigma, 96992), following the manufacturer’s instructions.

### Lentiviral Construction and Cell Transfection

All lentiviral constructs were prepared by Shanghai GeneChem Co., Ltd (Shanghai, China). Lentivirus GV287-SCD-1transfection was conducted following the manufacturer’s instructions. Generally, the AML-12 cells were infected with the GV287-SCD-1 or the control lentivirus GFP. The lentivirus vector and packaging plasmid mixes were transfected into AML-12 cells using Lipofectamine 2000 (Invitrogen Life Technologies, Carlsbad, CA, United States). Following 6–8 h incubation, the viruses were removed and replaced with a fresh medium. Three-days post-transfection, GFP expression was observed using a fluorescence microscope.

### Measurement of Cellular Triglyceride Content

The intracellular TG was extracted and purified using the method by Heider and Boyett (Heider and Boyett, 1978). The content of TG was determined with a biochemistry assay kit (Dongou Biology Technique Co. Ltd., Zhejiang, China), according to the manufacturer’s instructions.

### Quantitative Real-Time PCR

Custom primers were designed following our previous work (Feng et al., 2017). Primers were characterized by melting curve analysis, agarose gel electrophoresis, and DNA sequencing and synthesized at Eurofins MWG Operon (Huntsville, Alabama). Animal tissues were stored at −80 °C after soaking with RNA later (Qiagen, Valencia, CA). Total RNA isolated using the RNA Extraction kit (Cat# 9769, TaKaRa, Japan) was used to prepare cDNA with the RevertAid First Strand cDNA Synthesis kit (#K1622, Thermo). Quantitative qRT-PCR was performed on an iCycler iQ real-time detection system (Bio-Rad Laboratories) using SYBR Green (iQ SYBR Green Supermix, Bio-Rad Laboratories). Relative expression of each gene was calculated with β-actin or GAPDH as the housekeeping gene. Primer sequences were listed in Supplementary Table S3.

### Western Blot Analysis

Liver tissue and AML-12 cells were homogenized in a lysis buffer (150 mM NaCl, 1% Nonidet P- 40, 0.1% SDS, 50 mM Tris-HCl pH 7.4, 1 mM EDTA, 1 mM PMSF, 1x Roche complete mini protease inhibitor cocktail). The supernatants were collected after centrifugation at 10,000 g at 4°C for 15 min. Protein concentration was determined using a BCA protein assay kit (Beyotime Institute of Biotechnology, Jiangsu, China). Equal amounts of protein were separated by 10% SDS gel electrophoresis (SDS-PAGE) under denaturing and nonreducing conditions and transferred to a PVFD membrane. The membrane was blocked with 5% non-fat milk in TBST at room temperature for 1 h and then incubated with primary antibody at 4°C overnight (antibody information was presented in Supplementary Table S2.). After thrice washing in TBST, the blots were incubated with horseradish-coupled secondary antibody. The signals were visualized using an enhanced chemical luminescent (ECL) system (Pierce Biotechnology, Inc., Rockford, IL, United States) and recorded in the chemiluminescence imaging system (ChemiScope 3500 mini, Xiang Ying, China).

### Immunohistochemistry Staining

Paraffin-embedded liver tissues were prepared when sacrificed the mice. Briefly, after dewaxing, liver sections were submitted to antigen retrieval and further blocked using a blocking solution. The section was rinsed in PBS for 5 min, three times, and then incubated with the primary antibody (1:100 dilution) overnight at 4°C. Following washing in PBS, the sections were incubated with secondary antibody for 1 h.

### Statistical Analysis

Statistical analysis was performed using GraphPad Prism version 5.0 (GraphPad Software, La Jolla, CA). Data are expressed as mean ± standard deviation. Data were analyzed using the *t*-test or one-way analysis of variance and the least significant difference test, and *p* < 0.05 was considered statistically significant.

## Results

### The GC Combination, a Simplified Prescription of the ACG Recipe, Ameliorates the Hepatic Triglyceride Accumulation and Impairs the Elevated Liver Enzymes in Non-Alcoholic Fatty Liver Rats

**FIGURE 1:**
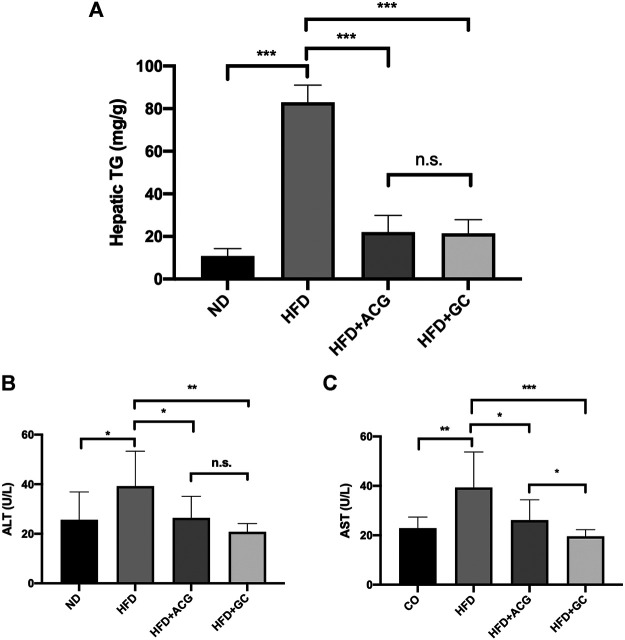
The GC combination ameliorates the hepatic triglyceride (TG) accumulation and impairs the elevated alanine aminotransferase (ALT) and aspartate transaminase (AST) levels in NAFL rats. **(A)** Hepatic TG content in rats (*n* = 9) **(B,C)** serum ALT and AST (*n* = 9). *p < 0.05, **p < 0.01, ***p < 0.001, n.s. = no significant.

Our previous data (Meng et al., 2016) has verified the therapeutic effects of the recipe composed of atractylodes macrocephala polysaccharide, chlorogenic acid, and geniposide (named ACG) on the experimental non-alcoholic fatty liver (NAFL). Here, the GC combination, only two components simplified from the ACG recipe, presented similar powerful ameliorations on hepatic TG accumulation (Figure 1A) as well as liver enzymes (Figures 1B,C) in NAFL rat models (HFD). What’s more, the GC combination showed more potent effects for reversing the pathologically elevated liver enzymes. The AST (aspartate transaminase) level of the GC group was even significantly lower than the ACG group level (Figure 1C).

### The GC Combination Markedly Ameliorates Lipid and Glucose Metabolism Serum Index and Normalizes the Liver Functional Enzymes

We next arranged mice experiments to elucidate whether the GC combination attenuates the HFD induced fatty liver. Meanwhile, for further identifying the individual effects of the G and C, the same dosage intervention separated from the GC combination was performed. Fifty mice were fed with a high-fat diet for 12 weeks and following with or without the respective pharmacological intervention for another 4 weeks.

**FIGURE 2:**
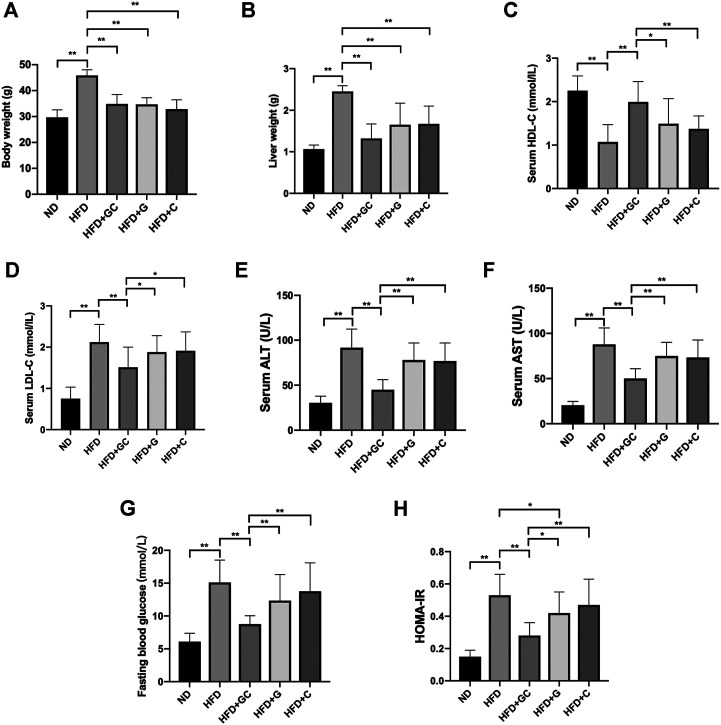
The GC combination reverses biochemical parameters in NAFL mice. The mice body weight **(A)**, liver weight **(B)**. The level of serum HDL-C **(C)** and LDL-C **(D)**, serum ALT **(E)** and AST **(F)**, fasting blood glucose **(G)**, and HOMA-IR **(H)** in the mice from each group (*n* = 10). *p < 0.05, **p < 0.01.

As shown before, the GC combination did not significantly affect food intake (Peng et al., 2018). After 4 weeks of treatment, the body weight and liver weight of mice in each treatment group were notably lower than those in the HFD group (Figures 2A,B). Meanwhile, the disordered serum HDL-C (high-density lipoprotein cholesterol) (Figure 2C) and LDL-C (low-density lipoprotein cholesterol) (Figure 2D) were normalized. The elevated serum ALT (alanine transaminase) and AST (Figures 2E,F) levels were also significantly reversed. Although the serum insulin level (data not shown) did not show a significant difference among the treatment groups, the GC combination strongly decreased the FBG (fasting blood glucose) (Figure 2G) and HOMA-IR (homeostatic model assessment-insulin resistance) (Figure 2H). Notably, comparing with the geniposide (G) or chlorogenic acid (C) alone, the GC combination showed a significant synergetic effect on normalizing the serum lipids and the blood glucose level, and reversing the increased AST and ALT induced by HFD.

### The GC Combination Markedly Ameliorates Hepatic Steatosis in Non-Alcoholic Fatty Liver Mice and Presents Significant Synergetic Effects than Each Component

**FIGURE 3:**
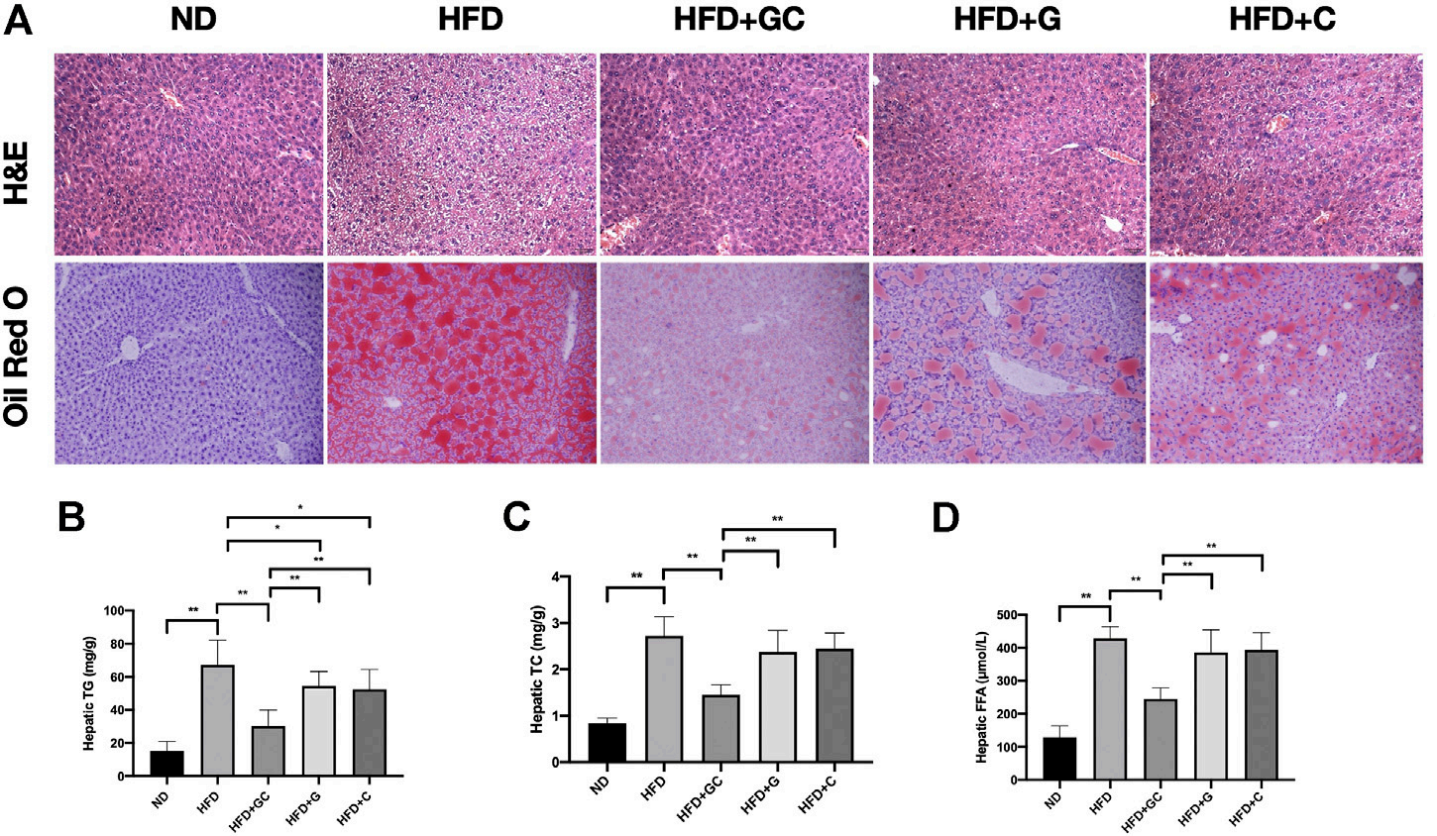
The GC combination ameliorates hepatic fat deposition induced by high-fat diet (HFD). **(A)** H&E and Oil Red O staining for liver sections (200 times of magnification). Hepatic TG, TC, and FFA content in each group (*n* = 10) **(B,C,D)**. *p < 0.05, **p < 0.01.

Attractively, grievous hepatic steatosis in NAFL mice was markedly attenuated after 4 weeks of the GC treatment, as demonstrated by H&E and Oil Red O staining (Figure 3A). With 16 weeks HFD feeding, steatosis and ballooning of hepatocytes and inflammatory cells infiltration were readily observed in lobules with hematoxylin and eosin (H&E) staining. Typical macrovesicular steatosis was showed in centrilobular regions. Consistently, as visualized in Oil Red O staining, dramatically increased lipid deposition occupied much of the cytoplasm of hepatocytes. However, after 4 weeks treating with the GC combination, the steatosis of hepatocytes is reversed, and rarer ballooning degeneration and inflammatory infiltration were showed. In line with the pathological changes in the liver section, the significantly reversed level of hepatic triglyceride (TG) (Figure 3B), total cholesterol (TC) (Figure 3C), and free fatty acid (FFA) (Figure 3D) were also presented in the GC treatment group. Again, even the pathological and biochemical ameliorations were also demonstrated in the G or C individual treatment group; the GC treatment showed remarkably synergetic effects on suppressing the hepatic lipid content.

### SCD-1 Is the Most Significant Differential Gene Down-Regulated by the GC Combination in NAFL Rats, and the Expression Is Consistently Suppressed in the NAFL Mice Model

**FIGURE 4:**
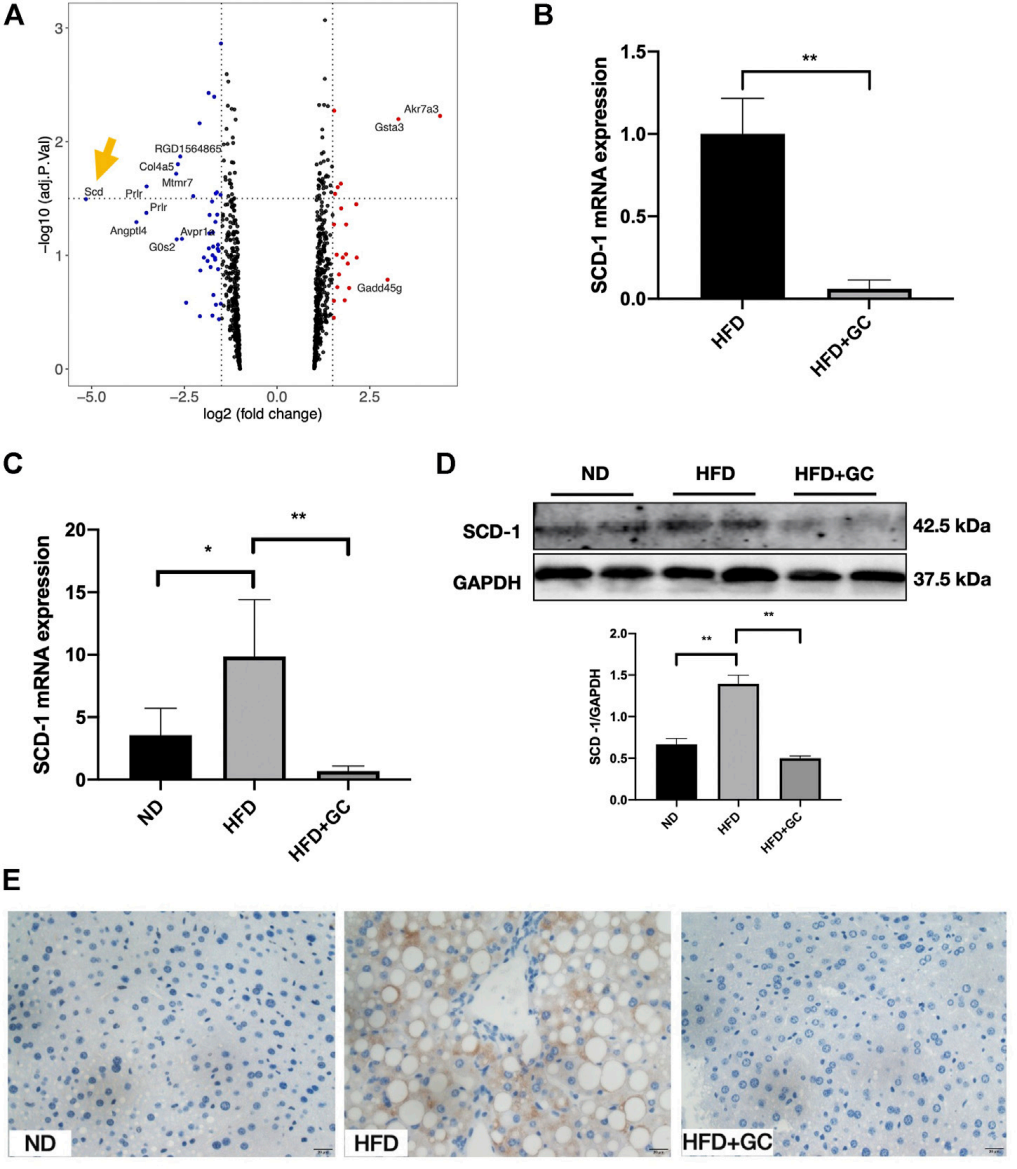
SCD-1 is the most significant differential gene highlighted in microarray and significantly down-regulated by the GC combination. **(A)** Volcano Plot for differential gene expression between HFD and HFD + GC group in the liver of rats (*n* = 3), logFC < 1 was deleted during the analysis. SCD-1 mRNA expression in rat **(B)** and mice **(C)** liver tissues was verified by real-time PCR analysis. SCD-1 protein expression in mice liver was detected by immunoblotting **(D)** and immunohistochemistry **(E)** (Brown area, 400 times of magnification), and the relative expression levels of proteins were corrected by GAPDH. *p < 0.05, **p < 0.01.

The microarray analysis was performed to search the potential therapeutic targets. The volcano plot highlighted that SCD-1 was the most attractive differential gene (Figure 4A) down-regulated (more than 5-fold down-regulated) by the GC combination in the liver tissue of NAFL rats (*n* = 3). The top five differential genes in rat liver tissue analyzed by microarray between the HFD and the GC combination treatment groups were presented in the Supplementary Table S1. For confirming the eye-catching result, the real-time PCR test was performed to identify the SCD-1 mRNA expression. The GC combination dramatically suppressed the SCD-1 mRNA expression (Figure 3B). Meanwhile, the SCD-1 transcriptional (Figure 3C) and translational (Figure 3D) levels in the liver of NAFL mice were also suppressed by the GC combination. Consistent with the immunoblotting result, SCD-1 was positively stained with the brown area around the ballooning lipid droplets in hepatocytes by immunohistochemistry staining (Figure 4E and Supplementary Figure S2), but the GC combination stably blunted the SCD-1 expression.

Since SCD-1 acts as a key mediator in the hepatic DNL process, SCD-1 might be the potential therapeutic target of the GC combination in improving hepatic lipid accumulation in NAFL rodent models.

### The GC Combination Markedly Ameliorates Cellular Steatosis and Reverses Up-Regulated SCD-1 in FFA-Exposed AML-12 Cells

**FIGURE 5:**
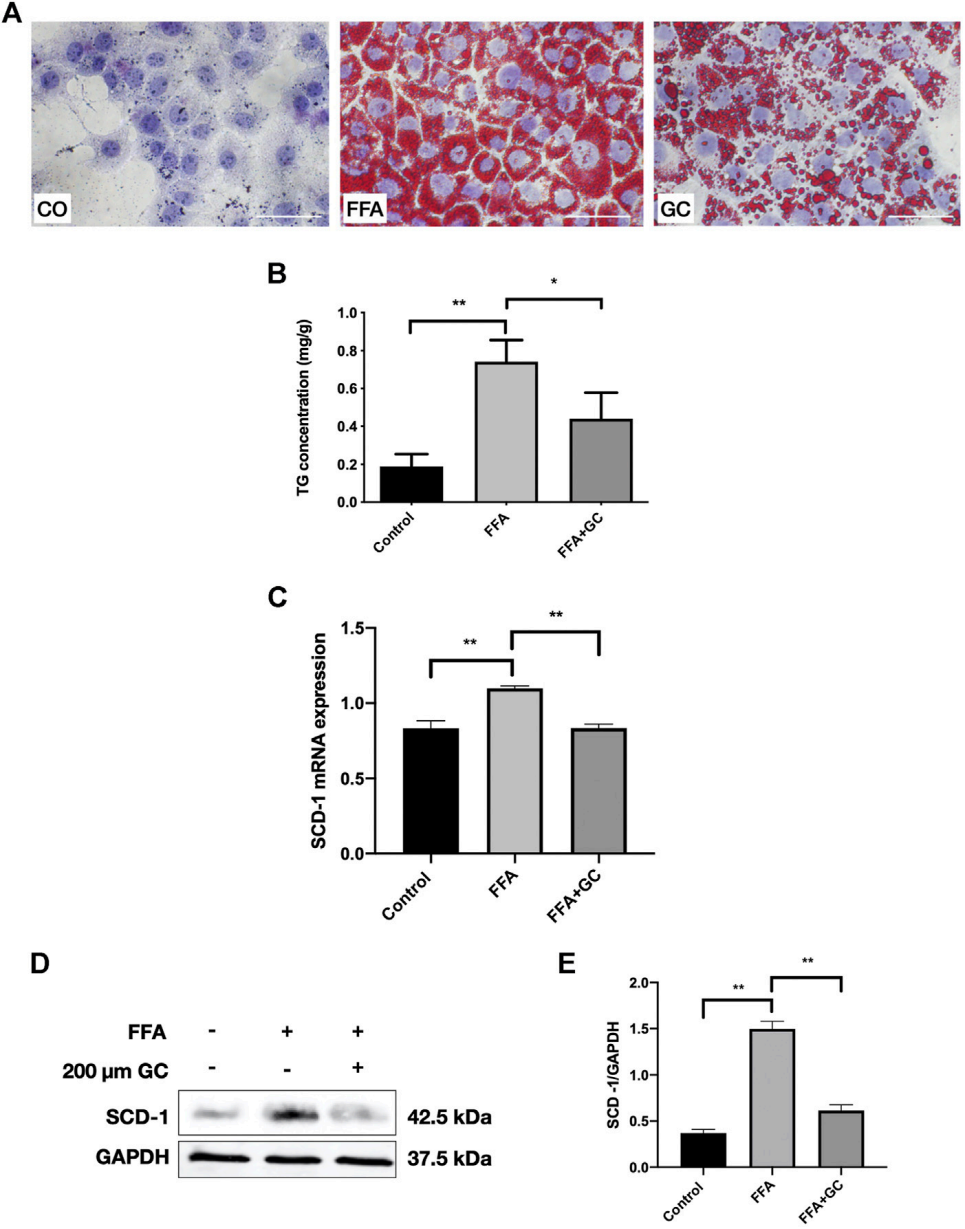
The GC combination ameliorates lipid droplet accumulation and reverses up-regulated SCD-1 induced by free fatty acid (FFA) in AML-12 cells. **(A**) Oil Red O staining of AML-12 cells (400 times of magnification). **(B)** intracellular TG content. **(C)** SCD-1 mRNA expression in cells was detected by qRT-PCR. **(D)** SCD-1 protein levels in cells were detected by immunoblotting, and the relative expression levels **(E)** of proteins were corrected by GAPDH. *p < 0.05, **p < 0.01.

To investigate the effect of the GC combination on FFA-induced lipid accumulation in AML-12 cells, we exposed the cells to a 0.3 mM FFA mixture with the presence or absence of the 200 μm GC combination. We first determined the concentration dependence of the cytotoxic effects of the GC in AML-12 cells by CCK8 assay. In 24 h, cell viability was not affected by the GC combination (Supplementary Figure S3A) up to 400 µm. Meanwhile, 48 h GC combination exposure (medium contained the GC combination was refreshed at 24 h), cell viability was not affected either (data not shown). In addition, 0.3–0.5 mM FFA mixture significantly increased cellular TG content in AML-12 cells (Supplementary Figure S3B). Hence, a 0.3 mM FFA mixture was used to induce the steatosis in AML-12 cells. Dramatically red-stained cytoplasmic lipid droplets accumulation induced by FFA mixture was confirmed by Oil-red O staining, but the accumulation was significantly reversed by the GC combination treatment (Figure 5A). Furthermore, the intracellular TG content was measured; the induced TG level was dramatically attenuated by the GC combination (Figure 5B). Meanwhile, the SCD-1 expression was activated, in transcriptional and translational levels, by FFA mixture exposure after 24 h. But the induced SCD-1 induction was significantly reversed by the GC combination (Figures 5C–E).

Together, *in vitro* data demonstrate that the GC combination significantly impaired the intracellular TG deposition, and it also suppressed the FFD induced SCD-1.

### The GC Combination Strongly Attenuates SCD-1 Expression in SCD-1 Overexpressed AML-12 Cells

**FIGURE 6:**
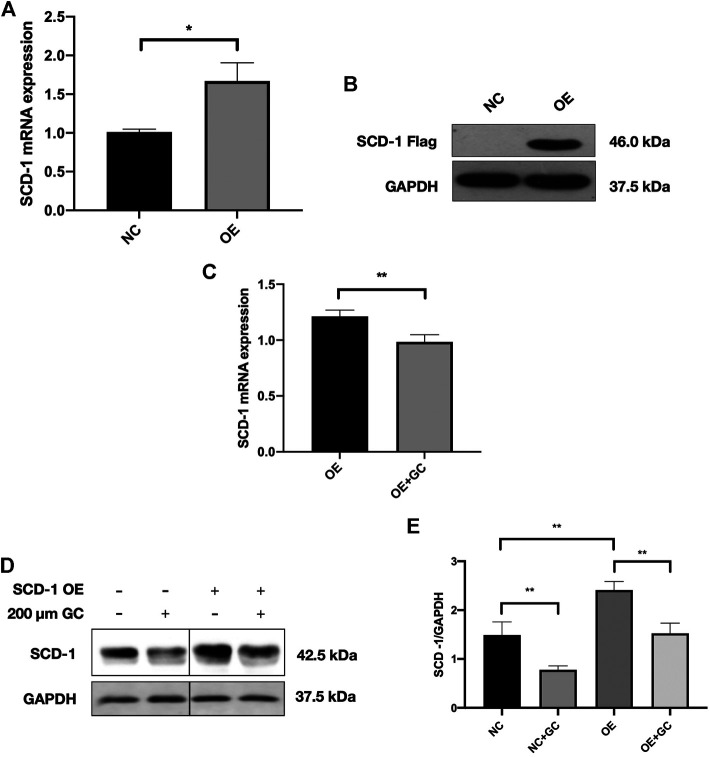
The GC combination dramatically suppresses the SCD-1 expression in SCD-1 over-expressed cell models **(A,B)** SCD-1 mRNA and protein levels in lentivirus transfected SCD-1 over-expressed AML-12 cells (OE). The same vector without the SCD-1 insert was used as control (NC). **(C)** SCD-1 mRNA expression in cells was detected by qRT-PCR. **(D)** SCD-1 protein levels in cells were detected by immunoblotting, and the relative expression levels of proteins **(E)** were corrected by GAPDH. *p < 0.05, **p < 0.01.

To further confirm whether the GC combination has a strong suppression effect on SCD-1, the lentivirus SCD-1 was used to stably overexpress SCD-1 in AML-12 cells and following with the presence or absence of the GC combination treatment. The overexpression of SCD-1 was initially confirmed by real-time PCR and western blotting tests. The significant up-regulated SCD-1 level stably presented in SCD-1 overexpressed AML-12 cells (OE), comparing with the non-insect vector control (NC) (Figures 6A,B). Subsequently, SCD-1 overexpressed cells were directly exposed to the GC combination treatment for 24 h. As expected, the GC combination significantly revered the lentivirus-mediated SCD-1 overexpression as before (Figures 6C–E).

**FIGURE 7:**
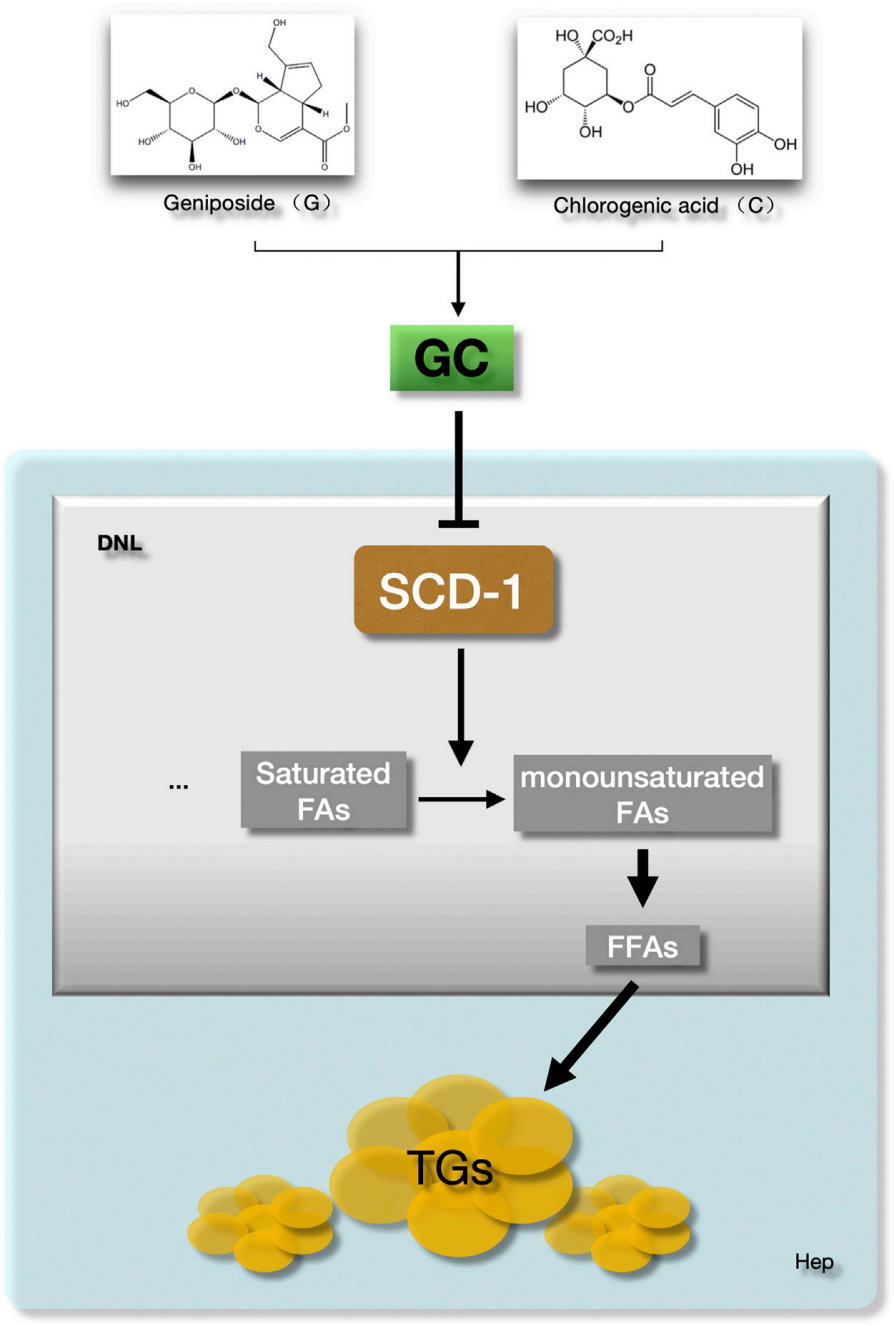
The GC combination ameliorates hepatic fat deposition by suppressing SCD-1. SCD-1 promotes the last stage of *de novo* lipogenesis to form the free fatty acid in hepatocytes. The GC combination was demonstrated to suppress the hepatic SCD-1expression, which highly impairs the DNL process and further attenuate the hepatic lipid deposition in NAFLD models. GC, Geniposide and chlorogenic acid combination, SCD-1, Stearoyl CoA desaturase-1, DNL, *de novo* lipogenesis, FFAs, Free fatty acids, TGs, Triglycerides.

Above all, the GC combination showed a powerful suppressing effect on hepatic SCD-1 expression and consequently triggered the hepatic DNL process impairment. These effects highly contribute to the amelioration of hepatic lipid accumulation in NAFLD models (Figure 7).

## Discussion

We here prove that the GC combination, composed of geniposide and chlorogenic acid, presents potent amelioration effects in treating non-alcoholic liver disease *in vivo* and *in vitro*. In the beginning, we focused on hepatic lipid accumulation changes in the presence or absence of the GC combination in the NAFL model and further compared the pharmacological effects with two individuals of the GC combination. After clarifying the ameliorating impacts, microarray analysis was performed to identify the potential therapeutic targets, and SCD-1 suppression was highlighted by microarray. Finally, two strategies for confirming the influence of the GC combination on SCD-1 in FFA triggered and lentivirus-mediated SCD-1 overexpressed cells were performed.

The pathogenesis of NAFLD is multifactorial, and its understanding still keeps incomplete. Nowadays, a multiple-hit hypothesis that implicates a myriad of factors acting in a parallel and synergistic manner in individuals with genetic predisposition is the more accepted understanding (Arab et al., 2018). A disturbed lipid and glucose homeostasis, increased lipid peroxidation, liver injury, and gut microbiota disorder triggered inflammation et al., drive the disease progression.

In our previous research, four weeks of the GC combination treatment significantly improve the hepatic inflammatory condition (Peng et al., 2018; Feng et al., 2017). HFD triggered hepatic IL-1β, TNF-a, LPB, TLR4 induction were significantly blunted. Meanwhile, the plasma LPS level and the F4/80, the biomarker of the Kupffer cell, were also dramatically down-regulated. And we also found that the GC combination had a significant impact on the gut microbiome structure and ameliorated colonic injury and incidence of inflammatory cell infiltration (Peng et al., 2018). The same, the hepatic glutathione level in NAFL rats was significantly up-regulated after the GC treatment, which highly elucidated that the GC combination presented the anti-oxidative stress function (Feng et al., 2017). To sum up, the GC combination displayed multi-therapeutic effects on NAFLD improvement, and these different targeting mechanisms highly interact with each other and make synergistic amelioration influences.

Importantly, lipid accumulation in hepatocytes is the key pathological feature of NAFLD. On the other hand, lipid metabolic disorder is the most direct etiology of this disease (Chen et al., 2019). Increased TG deposition in the liver reflects an input/output imbalance of hepatic FFA metabolism (Rotman and Sanyal, 2017). Our previous non-alcoholic steatohepatitis (NASH) with liver fibrosis mice models amply proved that a long term (30 weeks) of high fat, high carbohydrate diet crazily induced hepatic lipid deposition, even triggered liver fibrosis in mice liver (Xin et al., 2020). Here, mice continuously fed with a high-fat diet for 16 weeks ending up with NAFLD, characterized by markedly increased hepatocytes TG content, FFAs accumulation, and simultaneously companied by severe insulin resistance. Attractively, the GC combination presented a significant anti-lipogenic effect on the NAFL rodent’s liver and steatosis cell model. Moreover, compared with the G or C individual pharmacological intervention, the GC combination showed significantly stronger amelioration effects on hepatic lipid and glucose metabolism, and liver function normalization (ALT and AST). The synergetic effects were demonstrated by combining the G and C with a unique ratio (67.16:1). It should be noticed that the chlorogenic acid or geniposide individual treatment did not show any significant suppressing effects on the disordered hepatic TC or FFA accumulation. Still, this hepatic lipid deposition attenuation effect was dramatically enhanced with this unique combination.

Accordingly, three sources of FFAs are known to contribute to TG accumulation in the fatty liver. 25% come from the DNL process, the second major contributor of FFAs in hepatocytes (Donnelly et al., 2005; Chen et al., 2019). Besides, DNL is also increased under the condition of insulin resistance. And *vice versa*, the DNL rate would provide an early warning of the possible development of type 2 diabetes mellitus (T2DM) (Ameer et al., 2014). Here, the significantly decreased hepatic FFAs content and attenuated fasting blood glucose level *in vivo* strongly proved that the disordered hepatic DNL process went back to normal with the GC combination treatment.

During the DNL process, even several primary enzymes (e.g., acetyl-CoA carboxylase and fatty acid synthase) promote its going, and hepatic SCD-1 plays a critical role in the generation of monounsaturated FAs from saturated FAs, which is the last stage of DNL to form the FFAs (Chen et al., 2019). Increasing research demonstrated that suppressed SCD-1 expression and enzyme activity or SCD-1 knock out to make a great contribution to NAFLD amelioration (Ntambi et al., 2002; Biddinger et al., 2006; Miyazaki et al., 2007; Sampath et al., 2009). More importantly, SCD-1 activity was a predictor of the development of metabolic syndrome, and the expression of SCD-1 was associated with hepatic steatosis in humans (Warensjö et al., 2005; Poloni et al., 2015). In our present study, 16 weeks HFD robustly triggered the up-regulation of hepatic SCD-1 in NAFL rats and mice. Similarly, FFA also induced the SCD-1 expression in AML-12 cells.

Although the clinical burden of NAFLD is increasing rapidly, unfortunately, there are no FDA-approved effective drugs thus far. As a potential therapeutic target of NAFLD, SCD-1 inhibitor investigation attract attention (Rotman and Sanyal, 2017; Friedman et al., 2018; Chen et al., 2019). Again, the microarray assay provided us with a clue that SCD-1 might be a therapeutic target of the GC combination. Both *in vivo* and *in vitro* data strongly proved that the GC combination presents a potent suppressing effect on hepatic SCD-1 expression. Furthermore, this kind of solid suppression effect was demonstrated in the SCD-1 overexpressed cell model. Even SCD-1 expression was stably promoted by lentivirus transfection, and the GC combination undoubtedly reversed this up-regulation on transcriptional and translational levels. Above all, it has become clear that the GC combination reversed the hepatic lipid deposition in NAFL *in vivo* and *in vitro* models. More importantly, these effects are highly contributed to the robustly suppressed hepatic SCD-1 expression.

However, SCD-1, as the downstream regulator in hepatic lipid synthesis, is controlled by a sequence of upstream targets. Logically, suppressed SCD-1 either comes from the direct modulation of the GC combination or is influenced by its upstream targets, or both possibilities exist simultaneously. Accordingly, the known major upper-stream regulators of SCD-1 are leptin receptor (Lep-R) and sterol regulatory element-binding protein (SREBP)-1c (Biddinger et al., 2006). The leptin receptor bind with Leptin will further suppress SCD-1 expression. With the primary exploration, GC treatment significantly induced Lep-R expression *in vivo* and *in vitro* (data not shown). On the other hand, the n-terminal part of mature SREBP-1c will be translocated into the nucleus, which binds with the sterol regulatory elements (SRE) and further promotes the SCD-1 transcription (Ntambi, 1999; Ferré and Foufelle, 2007). Simultaneously, the GC combination blunted nSREBP-1c expression in NAFL models (data not shown). The exact mechanisms of the SCD-1 regulation with the GC combination will be deeply understood in our following research. The same, the specific mechanism(s) of the interaction between these two active ingredients in the GC combination to trigger these pharmacological synergetic impacts should be answered in the future.

Finally, natural active components attracted from herbs or food demonstrated promising therapeutic effects on NAFLD, such as EGCG (Chen et al., 2018) extracted from green tea and caffeine (J Martins, 2017) present multiple pharmacological effects in treating NAFLD. Except for making an effect on SCD-1, geniposide and chlorogenic acid have also been reported to ameliorate NAFLD by up-regulating PPAR-α (Liu et al., 2017b), GLP-1R expression (Zhang et al., 2016) et al. in hepatocytes. Nowadays, the current understanding of the pathophysiology of NAFLD provides a strong rationale for combination therapeutics for this disease (Rotman and Sanyal, 2017). The GC combination, to some extent, indicates a preliminary idea of combination therapy and also provides a promising choice of natural active components in treating NAFLD.

In summary, our data demonstrate that the GC combination presents inspiring pharmacological effects on ameliorating NAFLD, especially on impairing the lipogenesis in hepatocytes. Moreover, these therapeutic advantages are highly attributed to the dramatically suppressed hepatic SCD-1 expression and impaired *de novo* lipogenesis process.

## Data Availability

The raw data generated in this study can be found in NCBI using the accession number GSE87432.

## Abbreviations

NAFLD, non-alcoholic fatty liver disease; MAFLD, metabolic dysfunction-associated fatty liver disease; SCD-1, stearoyl-CoA desaturase-1; GC, geniposide and chlorogenic acid; QHD, Qushi Huayu Decoction; DNL, de novo lipogenesis; TG, triglyceride; TC, total cholesterol; FFA, free fatty acid; T2DM, type 2 diabetes mellitus; FBG, fasting blood glucose; IR, insulin resistance; ALT, alanine aminotransferase; AST, aspartate transaminase; HFD, high-fat diet; AML-12, alpha mouse liver 12.
