# Supplementary figures and images for: Geniposide and Chlorogenic Acid Combination Improves Non-Alcoholic Fatty Liver Disease Involving the Potent Suppression of Elevated Hepatic SCD-1
Source: Front Pharmacol. 2021 May 4;12:653641. doi: 10.3389/fphar.2021.653641 (PMC8129574; doi:10.3389/fphar.2021.653641)

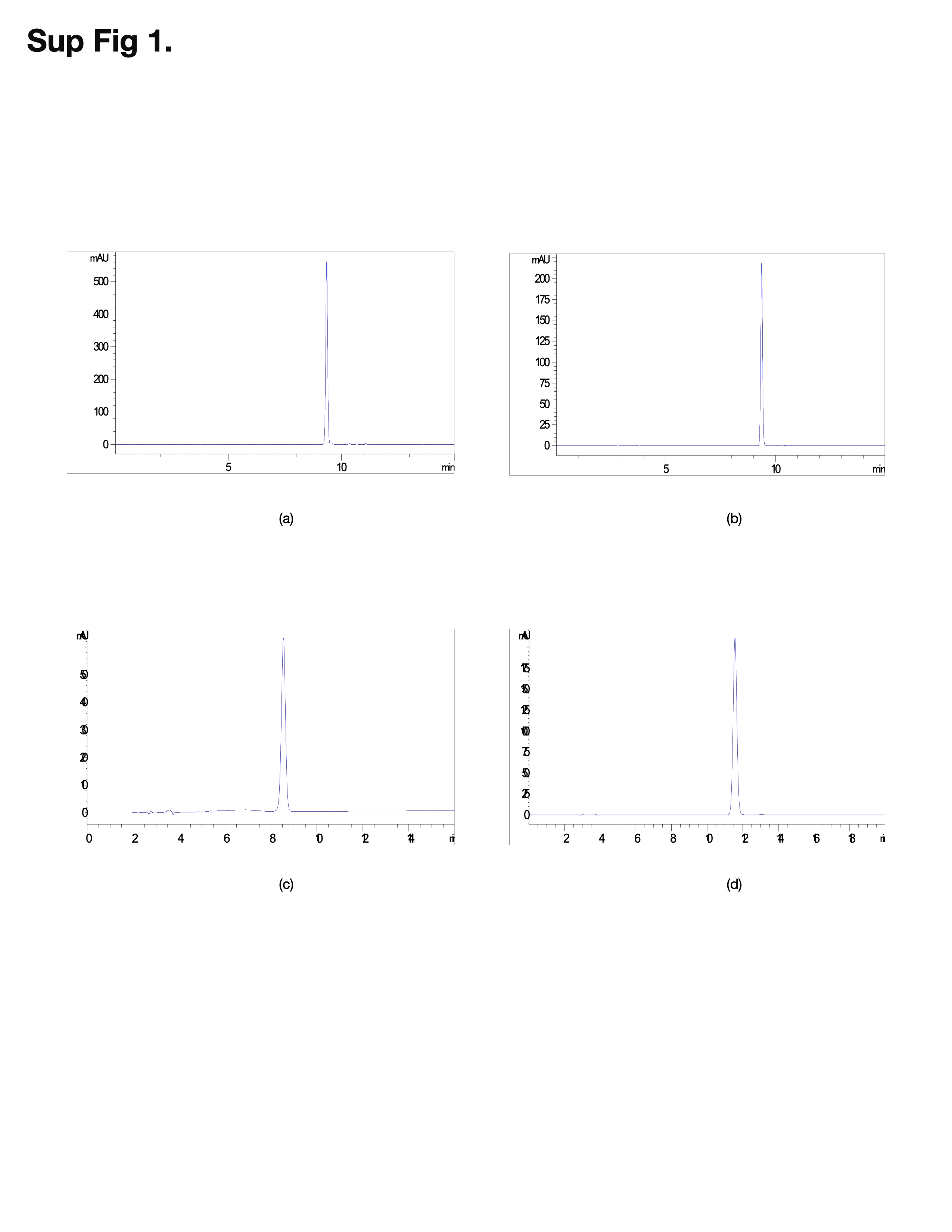

Supplement: Supplementary file 1 [file DataSheet1.zip › Image S1.jpg]

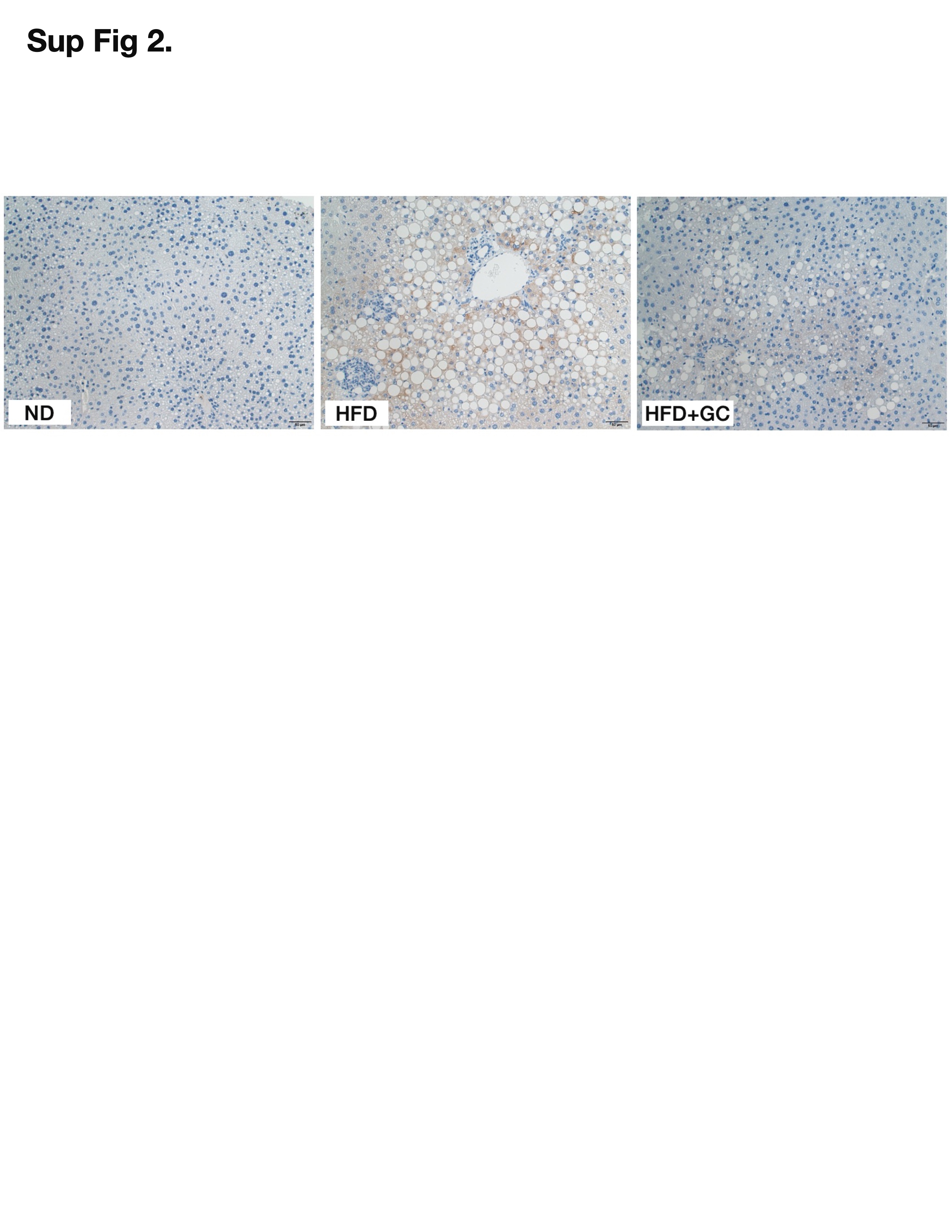

Supplement: Supplementary file 1 [file DataSheet1.zip › Image S2.jpg]

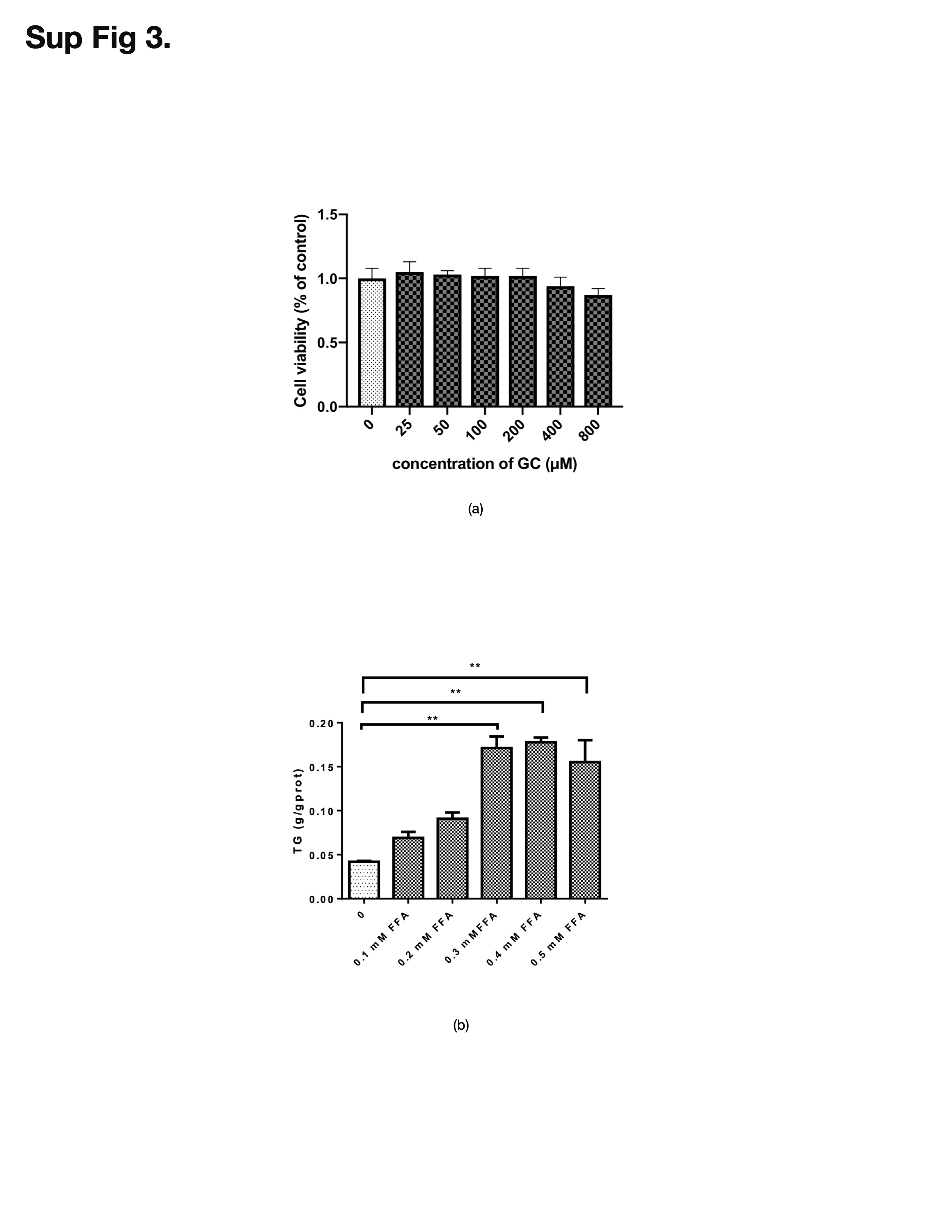

Supplement: Supplementary file 1 [file DataSheet1.zip › Image S3.jpg]

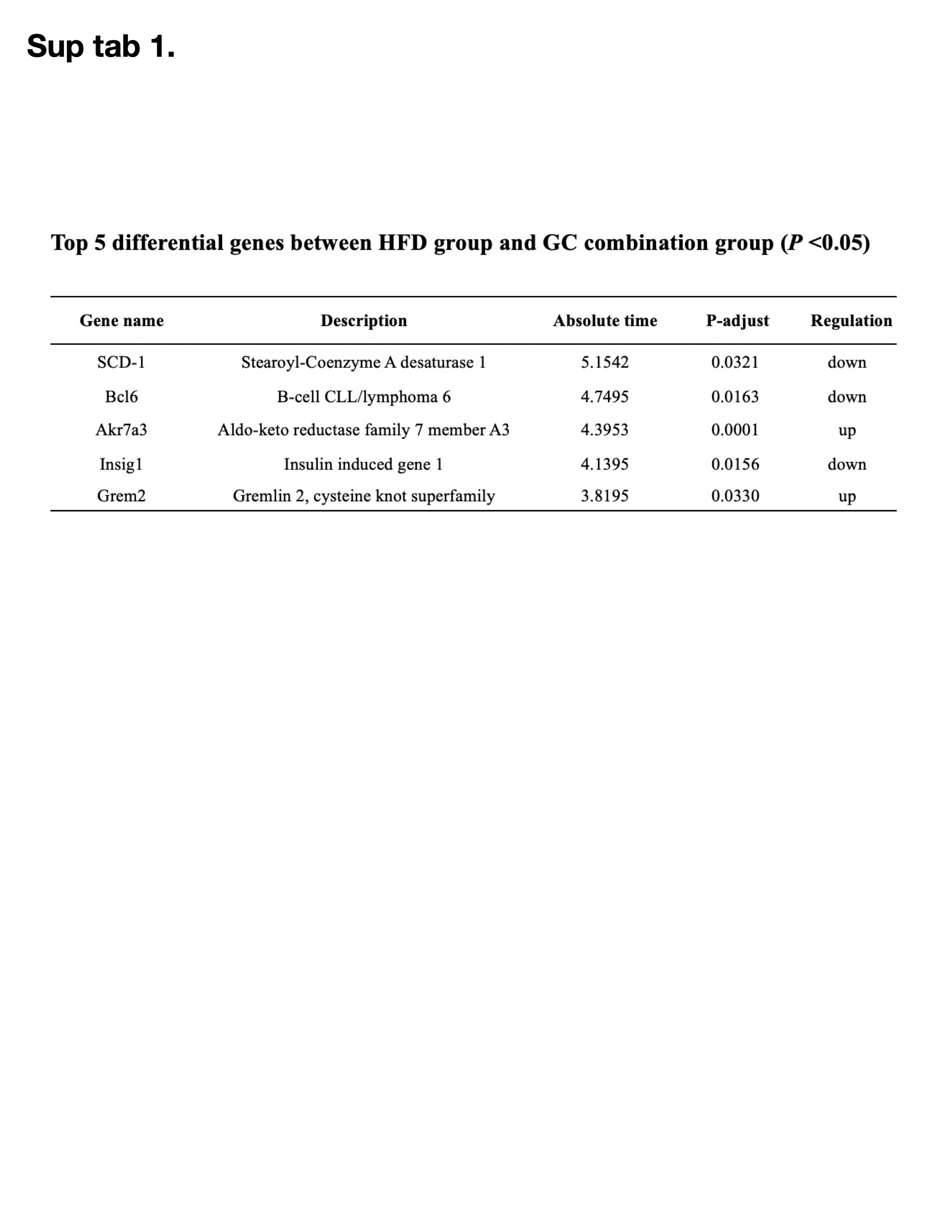

Supplement: Supplementary file 1 [file DataSheet1.zip › Table S1.jpg]

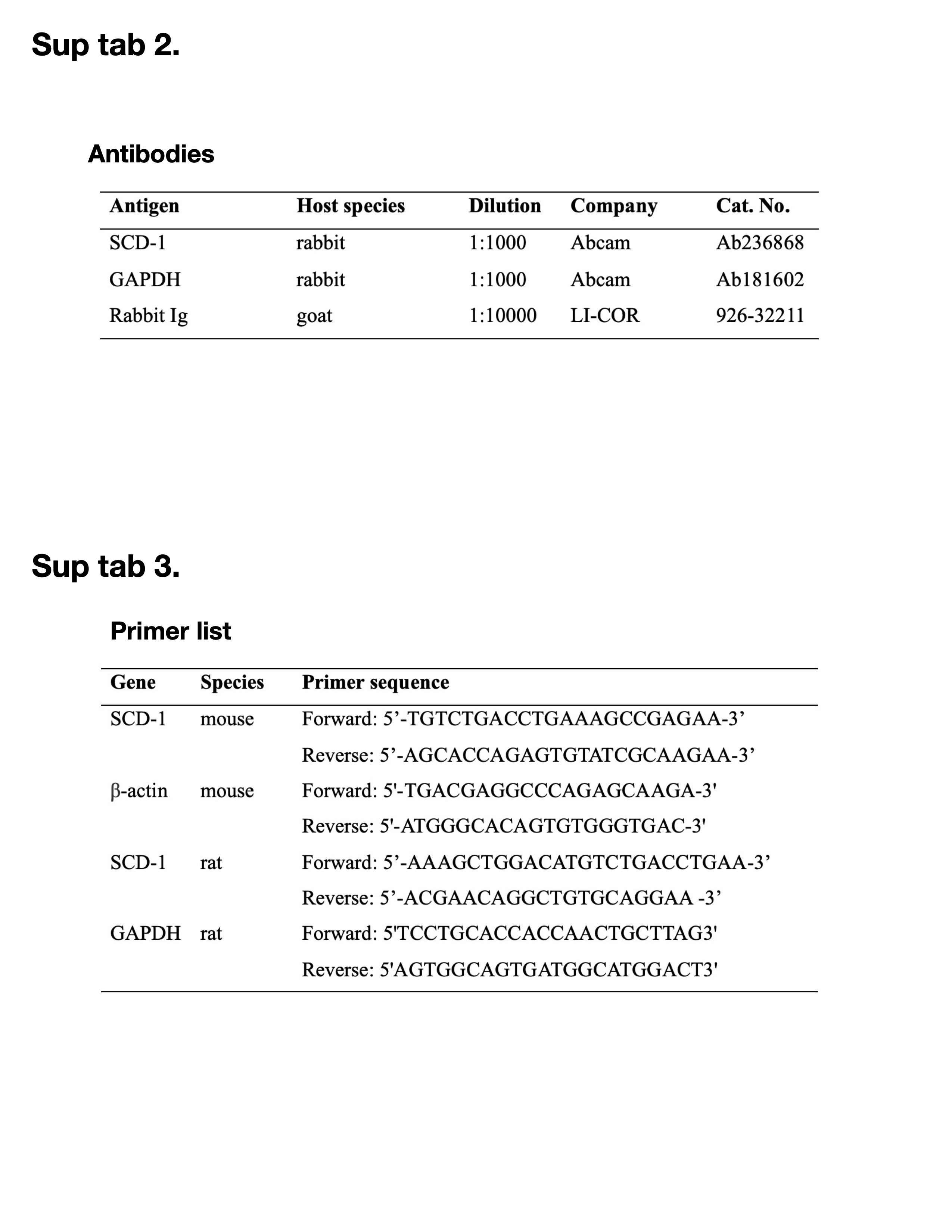

Supplement: Supplementary file 1 [file DataSheet1.zip › Tables S2 and S3.jpg]
